# IL-10, IL-6 and CD14 polymorphisms and sepsis outcome in ventilated very low birth weight infants

**DOI:** 10.1186/1741-7015-4-10

**Published:** 2006-04-12

**Authors:** R John Baier, John Loggins, Krishna Yanamandra

**Affiliations:** 1 Department of Pediatrics University of Manitoba WR 116 735 Notre Dame Avenue Winnipeg, Manitoba, R3E 0L8, Canada; 2 Department of Pediatrics Louisiana State University Health Sciences Center 1501 Kings Highway Shreveport, Louisiana 71130-3932, USA

## Abstract

**Background:**

Genetic variation in the innate immune system of the host may play a role in determining the risk of developing infection, as well as outcome from infection.

**Methods:**

Infectious complications were retrospectively determined in 293 (233 African-American (AA), 57 Caucasian and 3 Hispanic) mechanically ventilated very low birth weight (VLBW) infants (<1500 grams at birth) who were genotyped for the IL-6 -174 G/C, IL-10 -1082 G/A and CD14 -260 C/T single nucleotide polymorphisms (SNPs).

**Results:**

The IL-6 -174C allele was associated with an increased incidence of late blood stream infection (BSI) in AA but not Caucasian infants. In AA infants with the C allele the incidence of late BSI was 20/29 (69%) compared to 94/204 (46%) in homozygous GG infants (RR 2.6, 95% CI: 1.1–6.0, p = 0.021). The IL-10 -1082A allele was associated with an increased incidence of late BSI. One or more episodes of late BSI developed in 14 (35%) of 40 infants with the GG genotype, 71 (49%) of 145 infants with the GA genotype and 63 (58%) of 108 infants with the AA genotype (p = 0.036). Infants with the A allele (AA or GA genotypes) had an incidence of late BSI that was 134/253 (53%) compared to 14/40 (35%) in homozygous GG infants (RR 2.1, 95% CI: 1.04–4.19, p = 0.035). The CD14 -260 C/T SNP did not alter the overall risk for BSI in ventilated VLBW infants. Multiple BSI episodes were more common in the TT genotype group (CC: 17%, CT: 11%, TT: 30%, p = 0.022). This effect was due to the strong effect of the TT genotype on the incidence of multiple BSI in AA infants (CC: 15%, CT: 11%, TT: 39%, p = 0.003).

**Conclusion:**

The IL-6 -174 G/C, IL-10 -1082 G/A and CD14 -260 C/T SNPs may alter risk for BSI in ventilated VLBW infants.

## Background

Sepsis is a persistent and vexing problem in very low birth weight (VLBW) infants. The development of sepsis in this population has several adverse implications including; prolongation of hospitalization, development of chronic lung disease, adverse neurodevelopmental outcome and excess mortality [1]. The incidence of one or more episodes of late onset blood stream infection (BSI) in this population ranges from 25–30% with higher rates for infants with birth weights less than 1000 grams [2-5].

Genetic variation in the innate immune system of the host may play a role in determining the risk of developing and outcome from infection. Variation in the ability to recognize pathogens may influence the risk of infection. One of the primary molecules that function in recognition of pathogens is CD14. CD14 is expressed on phagocytic cells, and along with LPS-binding protein, it acts to transfer lipopolysaccharide (LPS) and other bacterial ligands to the Toll-like receptor 4 (TLR4)/MD-2 signaling complex [6]. The engagement of CD14 and LPS-binding protein during recognition of Gram-negative bacteria results in activation of a complex of innate host defense mechanisms. CD14 expression is influenced by a single nucleotide polymorphism (SNP) found in the proximal CD14 promoter. A C to T substitution 260 base pairs (bp) prior to the start codon diminishes binding of the inhibitory factor sp3 resulting in increased expression of the CD14 gene [7,8]. Homozygous carriers of the T allele have significant increases in both soluble and membrane-bound CD14 [9]. The CD14 -260 T allele may increase modify the risk for septic shock [10].

Variation in the magnitude of inflammatory response may also influence the risk of sepsis. Outcome from sepsis depends to a considerable degree on the host response. While an absent or diminished host response leads to overwhelming infection, an excessive response can lead to systemic inflammation and multiple organ failure [11-15].

Interleukin-6 (IL-6) is a proinflammatory cytokine that plays an important role in the host response to infection [16]. The C allele of IL-6 -174 G/C promoter polymorphism is associated with increased IL-6 production in newborn infants [17]. The IL-6 -174 G/C SNP has been inconsistently associated with altered risk for and outcome from sepsis in several studies [18,19].

Interleukin-10 (IL-10) is an anti-inflammatory cytokine produced by macrophages and T-helper-type II (TH2) lymphocytes that downregulates inflammatory mediator production by stimulated immune and epithelial cells [20-22]. Thus, IL-10 can potentially counterbalance the detrimental effects of excessive cytokine production in sepsis. The SNP at -1082 (G to A) modifies IL-10 secretion and may influence outcome in several disease states [23-25]. The IL-10 -1082 G/A SNP lies within a putative Ets transcription site and is associated (A allele) with lower IL-10 production in vitro [26]. The IL-10 -1082 SNP may modify the response to sepsis from a variety of organisms [23,27].

Variation in the IL-10, IL-6 and CD14 genes may be genetic factors influencing the development and outcome of sepsis in the premature newborn. The purpose of this study was to determine if there is a relationship between the IL-10 -1082 G/A, IL-6 -174 G/C and CD14 -260 C/T SNPs and risk for or outcome from sepsis in mechanically ventilated very low birth weight (VLBW) infants.

## Methods

The genomic DNA used for this case controlled study was extracted from archival tracheal aspirate (TA) pellets (259) or blood (34 patients) collected prospectively as part of an ongoing study of genetic factors in the development of complications of prematurity. The TAs that were used as a source of genomic DNA were originally collected as part of long term longitudinal studies examining cytokine concentrations and the development of CLD [28,29]. Infants were included in the study if they fulfilled the following inclusion criteria: birth weight less than 1500 grams, mechanical ventilation (MV) during the first week of life, complete clinical data on infectious outcome and genomic DNA sample that could be used for genotyping. Infants were excluded if complete data on outcome were not available or suitable DNA was not available. In order to compare the frequency of the various polymorphisms with normal term infants, control DNA was extracted from cord blood spots from a random sample of 168 African-American and 96 Caucasian term infants (performed independently as part of another study examining genotype and asthma). Consent was obtained from the parents of study infants to use the TAs and blood samples. The study was approved by the Institutional Review Board for Human Research at Louisiana State University Health Sciences Center in Shreveport.

Cultures for genital mycoplasmas were performed on TA samples collected in the first few days of life. Clinical and outcome data were abstracted from the clinical record and included information on respiratory outcome, survival and development of complications of prematurity. The results of all cultures of blood, tracheal aspirates and cerebrospinal fluid were recorded from the patients' charts. Tracheal aspirates were obtained twice a week in all intubated patients according to unit policy. Infants were evaluated for sepsis at the discretion of the clinical staff when signs and symptoms compatible with sepsis developed. Generally, 2 blood cultures from separate sites were obtained when assessing infants for blood stream infection. For the purposes of this study any positive blood culture was considered a blood stream infection (bacteremia or fungemia). Blood stream infections (BSI) were divided into early if the culture was obtained during the first 3 days of life, and late (nosocomial) if it was obtained thereafter. Nosocomial pneumonia was diagnosed when there was radiological evidence of a new pulmonary infiltrate and the blood and endotracheal aspirate culture grew the same organism. Isolation of an organism from a TA culture in an infant greater than 3 days of age without a positive blood culture or a change in chest radiograph was considered colonization. Sepsis mortality was defined as mortality during an acute episode of sepsis.

### Laboratory methods

Isolation of total DNA from blood or TA pellets was performed using the QIAmp DNA Mini kits™ (Qiagen Incorporated, Chatsworth, CA). Briefly, TA pellets were suspended in 200 μl of sterile phosphate buffered saline by vigorous vortexing, then digested with proteinase K and applied to silica gel spin columns. Columns were washed with the manufacturer's supplied buffers and the total DNA was eluted in 200 μl elution buffer. Blood (200 μl) was extracted similarly to the TA pellets.

The IL-10 -1082 G/A and IL-6 -174 G/C SNPs were genotyped using published allele specific PCR methods [25,30]. The CD14 -26/0 CT SNP was genotyped using a published restriction fragment length polymorphism method [9].

### Data analysis

Data analysis consisted of comparing the incidence of infections and their complications between the various genotypes. All statistical analysis was performed using SPSS for Windows version 6.0 (SPSS Inc., Chicago, IL). Differences in frequencies of complications were assessed by Chi square. ANOVA or the Student t-test was used to assess normally distributed variables where appropriate. The Wilcox Rank Sum test was used for analysis of factors that were not normally distributed. A probability value of less than 0.05 was considered statistically significant. The data are presented as mean ± standard error of the mean (SEM).

## Results

Two hundred and ninety-three (293) patients had complete culture and clinical information. Two hundred and thirty-three (79%) were African-American, 57 (20%) were Caucasian and 3 (1%) were Hispanic. Mean gestational age and mean birth weight of the study population were 26.7 ± 0.1 weeks and 906 ± 13 grams, respectively. Male: female ratio was 176:117. All patients required MV at birth and 264 (90%) infants were treated with exogenous surfactant therapy (Survanta^®^, Ross Products Division, Abbott Laboratories, Columbus, OH). One hundred and fifty eight (54%) infants were oxygen dependent at 28 days and 64 (22%) were oxygen dependent at 36 weeks PCA. There were 40 (14%) patients who died during their initial hospitalization (25 before 28 days of age and 15 after 28 days). Late onset BSI was the major cause of late mortality in ventilated VLBW infants with 14/15 deaths occurring after 28 days of age directly attributable to BSI (RR 15.3, 95% CI 2.0–117.9; p < 0.001).

One patient had positive blood cultures (Group B streptococci) during the first 3 days of life (early BSI) and 147 (50%) had one or more episodes of late (nosocomial) BSI. Coagulase negative staphylococci (CONS) were the organisms most commonly isolated from blood cultures. Seventy-one (24%) infants had one or more episodes of bacteremia/fungemia other than from CONS and 46 (15.7%) had multiple episodes of bacteremia or fungemia. Organisms causing nosocomial BSI (bacteremia/fungemia) for the study population are shown in Table 1. There were 15 (5%) deaths directly attributable to sepsis. Fourteen of 15 sepsis related deaths were associated with a non-CONS BSI (p < 0.001, RR = 59; 95% CI 7–421).

Endotracheal (ET) cultures obtained during the first 3 days of life grew *Ureaplasma urealyticum *in 86 (29%), *Mycoplasma hominis *in 35 (12%) and other bacteria in 12 (4%) infants. Subsequent bacterial colonization of the endotracheal tube (ETT) was detected in 145 (49%) patients. After birth, ETTs were most commonly colonized with CONS, less frequently with other organisms (Table 1). Nosocomial pneumonia was diagnosed in 22 (7%) infants (Table 1).

### IL-6 -174 G/C polymorphism and infectious complications

The frequency of the IL-6 -174 C allele in the study population was 0.128. The frequency of the C allele was significantly less in African-American (0.07) than in Caucasian infants (0.385) (p < 0.001). Two hundred and four (87.6%) African-American infants were homozygous GG, 27 (11.6%) were heterozygous GC and 2 (0.8%) were homozygous CC. In Caucasian infants the distribution of genotypes was: 19 (33.3%) GG, 32 (56.1%) GC and 6 (10.5%) CC. The 3 Hispanic infants were all GG. There were no differences in the frequency of the IL-6 -174C allele between study infants and ethnically matched control (term) infants [see Additional File 1].

Birth weight, gestational age, gender, TA isolation of Uu or Mh and need for surfactant replacement did not differ among genotype groups in either Caucasian or African American infants. Because the frequency of the IL-6 -174C allele was significantly different between ethnic groups, the effects of this SNP on infectious complications were analyzed separately for Caucasian and African-American infants.

The IL-6 -174C allele was associated with an increased incidence of late BSI in African-American infants. One or more episodes of late BSI developed in 94 (46%) of 204 infants with the GG genotype, 18 (67%) of 27 infants with the GC genotype and 2 (100%) of 2 infants with the CC genotype (p = 0.046). In infants who had the C allele (CC or GC genotypes) the incidence of late BSI was 20/29 (69%) compared to 94/204 (46%) in infant who were homozygous GG (RR 2.6, 95% CI: 1.1–6.0, p = 0.021). There were no significant differences in organism-specific BSI rates among genotype groups (Table 2). Neither the rates of non-CONS BSI (GG: 24%, GC: 44%, CC: 0%; p = 0.116) or multiple BSI episodes (GG: 15%, GC: 19%, CC: 0%, p = 0.752) were different among genotype groups.

The IL-6 -174 G/C polymorphism had no effect on sepsis-related mortality in African-American infants. Overall sepsis mortality was (GG: 5%, GC: 4%, CC: 0%; p = 0.916). The rate of sepsis mortality for African-American infants with late BSI was (GG: 11%, GC: 6%, CC: 0%; p = 0.717).

The rate of colonization of ETT tubes and incidence of nosocomial pneumonia were not affected by the IL-6 -174 G/C SNP. Nosocomial pneumonia rates were: (GG: 8%, GC: 7%, CC: 0%; p = 0.909). ETT colonization rates were 25/54 (46%) for infants with the GG genotype, 68/128 (53%) GC genotype, and 52/113 (46%) CC genotype (p = 0.489). CONS were the most common bacteria colonizing the ET tube in both groups. Organism-specific colonization rates were not different among genotype groups.

In contrast to that observed in African-American infants, the IL-6 -174 G/C SNP had no effect on the incidence of late BSI in Caucasian infants. One or more episodes of late BSI developed in 12 (63%) of 19 infants with the GG genotype, 16 (50%) of 32 infants with the GC genotype and 3 (50%) of 6 infants with the CC genotype (p = 0.643). The incidence of fungal BSI was increased in Caucasian infants with CC genotype (2/6 (33%)) compared to infants with the GG (5%) and GC (3%) (p = 0.027) (p = 0.008 comparing CC vs. GC and GG). There were no other significant differences in organism specific BSI rates among genotype groups (Table 2). Neither the rates of non-CONS BSI (GG: 21%, GC: 28%, CC: 33%; p = 0.827) nor multiple BSI episodes (GG: 21%, GC: 13%, CC: 33%, p = 0.415) were different among genotype groups.

The IL-6 -174 G/C SNP had no effect on sepsis related-mortality in Caucasian infants. Overall sepsis mortality was (GG: 5%, GC: 6%, CC: 17%; p = 0.614). The rate of sepsis mortality for Caucasian infants with late BSI was (GG: 8%, GC: 7%, CC: 33%; p = 0.383).

The rate of colonization of ETT tubes and incidence of nosocomial pneumonia in Caucasian infants were not affected by the IL-6 -174 G/C polymorphism. Nosocomial pneumonia rates were: (GG: 5%, GC: 7%, CC: 0%; p = 0.813). ETT colonization rates were: 11/19 (58%) for infants with the GG genotype, 14/32 (44%) GC genotype and 3/6 (50%) CC genotype (p = 0.620). Organism-specific colonization rates were not different among genotype groups.

The incidences of necrotizing enterocolitis (NEC), CSF infection and infections in other sites were not different among genotype groups in either African-American or Caucasian infants.

### IL-10 -1082 G/A polymorphism and infectious complications

The frequency of the IL-10 -1082A allele in the study population was 0.62. The frequency of the A allele was similar between African-American (0.63) and Caucasian infants (0.55) (p = 0.191). Forty (13.7%) infants were homozygous GG, 145 (47.3%) were heterozygous GA and 108 (36.9%) were homozygous AA. Distributions of genotypes were not significantly different between African-American and Caucasian infants (p = 0.088). There were no differences in the frequency of the IL-10 -1082 A allele between study infants and ethnically matched control (term) infants [see Additional File 1]. However, the frequency of the A allele was significantly different between Caucasian and African-American controls [see Additional File 1].

Birth weight, gestational age, gender, TA isolation of Uu or Mh and need for surfactant replacement were not different among genotype groups in either Caucasian or African American infants.

The IL-10 -1082A allele was associated with an increased incidence of late BSI in our study infants. One or more episodes of late BSI developed in 14 (35%) of 40 infants with the GG genotype, 71 (49%) of 145 infants with the GA genotype and 63 (58%) of 108 infants with the AA genotype (p = 0.036). In infants who had the A allele (AA or GA genotypes) the incidence of late BSI was 134/253 (53%) compared to 14/40 (35%) in infant who were homozygous GG (RR 2.1, 95% CI: 1.04–4.19, p = 0.035). Although they were not statistically significant, similar trends were observed when African-American and Caucasian infants were analyzed separately [see Additional File 2, Additional File 3, Additional File 4]. There were no significant differences in organism-specific BSI rates among genotype groups (Table 3). The rates of non-CONS BSI (GG: 15%, GA: 40%, AA: 26%; p = 0.676) or multiple BSI episodes (GG: 10%, GA: 16%, AA: 18%, p = 0.528) were not different among genotype groups. There was a trend towards increased incidence of non-CONS BSI in infants with the IL-10 -1082A allele (5/40 infants with GG compared to 66/253 infants GA/AA; p = 0.062).

The IL-10 -1082 G/A SNP had no effect on sepsis-related mortality. Overall sepsis mortality was: GG: 3%, GA: 6%, AA: 6% (p = 0.721). Sepsis mortality in infants with late BSI was: GG: 7%, GA: 11%, AA: 10% (p = 0.877).

The IL-10 -10814 G/A SNP did not affect ETT colonization or incidence of nosocomial pneumonia. Nosocomial pneumonia rates were: (GG: 11%, GA: 6%, A: 9%; p = 0.620). ETT colonization rates were: 18/40 (45%) for infants with the GG genotype, 74/145 (51%) GA genotype, and 53/108 (49%) AA genotype (p = 0.791). Organism-specific colonization rates were not different among genotype groups.

The incidences of necrotizing enterocolitis (NEC), CSF infection and infections in other sites were also not different among genotype groups in either African-American or Caucasian infants.

### CD14 -260 C/T polymorphism and infectious complications

The frequency of the CD14-260T allele in the study population was 0.33. The frequency of the T allele was similar between African-American (0.32) and Caucasian infants (0.39) (p = 0.151). One hundred and thirty-two (45%) infants were homozygous CC, 128 (44.2%) were heterozygous CT and 33 (11.3%) were homozygous TT. The frequency of the CD24 -260T allele was significantly greater in Caucasian term infants than in Caucasian VLBW and African-American term infants [see Additional File 1]. Birth weight, gestational age, gender, TA isolation of Uu or Mh and need for surfactant replacement were not different among genotype groups in either Caucasian or African American infants.

The CD14 -260CT SNP had no effect on the overall incidence of late BSI. One or more episodes of late BSI developed in 63 (48%) of 132 infants with the CC genotype, 66 (52%) of 128 infants with the CT genotype and 19 (58%) of 33 infants with the CC genotype (p = 0.570). However, there were significant differences in organism-specific rates of BSI between African-American and Caucasian infants (Table 4). In particular, in African-American infants, Gram-negative BSI was associated with the CC genotype (Incidence of infection CC: 12%, CT: 2% and TT: 9%, p = 0.020). There was a trend for non-CONS BSI to be more frequent in infants with the TT genotype (CC: 24%, CT: 20%, TT: 39%; p = 0.074). Multiple BSI episodes were more common in the TT genotype group (CC: 17%, CT: 11%, TT: 30%, p = 0.022). This effect was due to the strong effect of the TT genotype on the incidence of multiple BSI in African-American infants (CC: 15%, CT: 11%, TT: 39%, p = 0.003).

The CD14 -260 CT SNP had no effect on sepsis-related mortality. Overall sepsis mortality was (CC: 6%, CT: 4%, TT: 6%; p = 0.709). Sepsis mortality in infants with late BSI was (CC: 10%, CT: 8%, TT: 20% (p = 0.741).

The rate of colonization of ETT tubes and incidence of nosocomial pneumonia were not affected by the CD14 -260 CT SNP. Nosocomial pneumonia rates were: (CC: 11%, CT: 5%, CC: 7%; p = 0.190). ETT colonization rates were: 66/132 (50%) for infants with the CC genotype, 58/128 (45%) CT genotype, and 21/33 (64%) TT genotype (p = 0.170). Organism-specific colonization rates were not different among genotype groups.

The incidences of necrotizing enterocolitis (NEC), CSF infection and infections in other sites were not different among genotype groups in either African-American or Caucasian infants.

### Interactions between IL-10 -1082 G/A and IL-6 -174 G/C and incidence of sepsis

Since the IL-10 -1082A and the IL-6 -174C alleles were associated with an increased incidence of late BSI, the interaction of these two polymorphisms were examined on this and other outcomes. The effect of carriage of neither, either or both of the IL-10 -1082A and the IL-6 -174C alleles and IL-10 -1082:IL-6 -174 haplotypes was examined. Eight of the 9 possible haplotypes were present in our patient population. The IL-10 -1082 GG: IL-6 -174CC haplotype was not observed. Carriage of both the IL-10 -1082A and the IL-6 -174C alleles was associated with the greatest risk of late BSI in the overall population (p = 0.073) (Table 5). The incidences of CONS BSI, non-CONS BSI, multiple BSI and sepsis-related mortality paralleled that of the overall late BSI rate but were not significant (Table 5). This increase in risk was seen primarily in African-American infants (p = 0.031) [see Additional File 2 and Additional File 3]. No significant trend between haplotype and risk for sepsis was observed, although the highest incidence of late BSI was in infants with the IL-10 -1082AA: IL-6 -174 CC haplotype [see Additional File 5]. In multivariate analysis (logistic regression), carriage of the IL-10 -1082 A allele (p = 0.035) but not the IL-6 -174 C allele (p = 0.152) was a significant predictor of late BSI.

## Discussion

Genetically determined variation in the magnitude of inflammatory response may play a role in determining outcome from serious infections. IL-6 is a pro-inflammatory cytokine associated with the development of shock and mortality from sepsis [31]. Therefore genetic variants that influence production of this cytokine may have important implications in the development of and outcome from sepsis in the preterm neonate. The frequency of the IL-6 -174 C allele in both Caucasian and African-American VLBW infants was similar to control term infants and as described in other populations [19,32,33]. This suggests that this polymorphism has no effect on the incidence of prematurity or on the need for ventilation at birth. We found that the IL-6 C allele was associated with an increased risk for late BSI (all organisms) in African-American but not Caucasian infants. However, the incidence of fungal BSI was greatly increased in Caucasian infants with CC genotype compared to infants with GG or GC. This suggests that the IL-6 -174CC genotype may be a risk factor for fungal sepsis in specific ethnic groups. However, because there were relatively few Caucasian patients with the IL-6 -174 CC genotype (6), caution must be exercised in interpreting this finding. Further studies are needed to confirm this association.

Earlier studies had suggested that the IL-6 -174 G/C polymorphism was either not associated with increased risk of sepsis, or that VLBW infants who were homozygous GG were at increased risk of Gram positive sepsis [18,34]. However, the infants in these studies were all or predominately Caucasian. Ethnic differences and small numbers of Caucasian infants in our study may account for some of the discrepancies. In addition, our infants were significantly smaller, less mature and required mechanical ventilation, factors that significantly increase risk of infection and may overwhelm any effect of this polymorphism. There was no effect of the IL-6 -174 G/C polymorphism on mortality-related sepsis. The Ahrens study did not address the effect of polymorphisms on sepsis mortality. In adults, the GG genotype was associated with increased survival in sepsis, but had no effect on the incidence of sepsis (intensive care setting) [19].

The role of the IL-6 -174 G/C polymorphism on IL-6 production is unclear. The IL-6 -174 C allele has been associated with decreased transcriptional activity in response to LPS and IL-1α [33]. The effects of this polymorphism, however, are more complex and may be stimulus dependent, cell line dependent and different *in vivo *from *in vitro*. *In vitro *IL-6 production in LPS-stimulated mononuclear cells is higher in the CC genotype in newborn infants [17]. Following coronary artery bypass surgery the C allele is associated with increased plasma IL-6, whereas following vaccination the G allele is associated with increased plasma IL-6 [35-37]. This complexity is further compounded by additional functional polymorphisms that are in linkage disequilibrium with the -174 SNP [35,36].

IL-10 downregulates inflammatory mediator production by stimulated immune and epithelial cells. Production of IL-10 can have a potentially beneficial effect by dampening excess inflammatory mediator production in sepsis. Excessive IL-10 production can, however, lead to the phenomenon of immunoparalysis by inhibiting the response of macrophages to pathogenic bacteria. The frequency of the IL-10 -1082 A allele in our ventilated VLBW infants is similar to that in control term infants and to that described in the literature [27,34,38-40]. This suggests little effect of this allele on either the incidence of preterm birth or the need for mechanical ventilation at birth. Our results are consistent with those of Kalish et al. [41], in which the IL-10 -1082 SNP was not associated with risk of preterm birth.

The IL-10 -1082A allele (lower IL-10 production) was associated with a two-fold increase in the incidence of late BSI in ventilated VLBW infants. Although not statistically significant, there was a trend towards increased non-CONS BSI (associated with higher mortality and morbidity) in infants with the A allele. This suggests that a more robust inflammatory response may protect the host from invasive disease. There was, however, no effect on sepsis-related mortality. The effect of the IL-10 -1082 G/A polymorphism on the incidence and outcome from infectious disease has been contradictory and may be organism-specific or vary according to ethnicity. There was no effect of the IL-10 -1082 G/A polymorphism on the incidence of invasive meningococcal disease, but disease severity and mortality were associated with the AA genotype [27]. In contrast, the G allele was associated with the development of septic shock in pneumococcal sepsis and severity of the systemic inflammatory response in community acquired pneumonia, whereas there was no association between genotype and risk of infection [23,42]. In the VLBW infant, an earlier smaller study showed no effect of the IL-10 -1082 G/A polymorphism on development of sepsis [34].

The role of polymorphic variation in the IL-10 gene and IL-10 production is unclear. Variation in IL-10 production in relation to the IL-10 -1082 SNP is affected by the nature of inducing stimulus and association of other polymorphisms [26,39,40,43-46]. The IL-10 -1082 SNP is in linkage disequilibrium with two other SNPs (-819 C/T and -592 C/A) and appears in three haplotypes [26,47,48]. The GCC haplotype (G at position -1082, C at position -819, C at -592) is associated with high IL-10 secretion, while the ACC and ATA haplotypes are associated with intermediate and low IL-10 secretion respectively [49]. The -2849 A/G SNP also affects IL-10 production functionally and should be studied [40,43,44].

Genetic variation in the ability to recognize and respond to invading organisms may also significantly influence the development of and outcome from infection. CD14 is an important component of innate immunity and plays a role in recognizing both Gram negative and positive organisms. We found that the CD14 -260 C/T SNP did not alter the overall risk for BSI in ventilated VLBW infants. However, the CC genotype was associated with an increased incidence of Gram negative BSI in African-American infants, suggesting a potential role for this polymorphism in determining risk for certain types of infection or in specific ethnic groups. In addition, multiple BSI episodes were more common in infants with the TT genotype owing to the strong effect of the TT genotype on the incidence of multiple BSI in African-American infants. Ahrens et al. demonstrated no association between this SNP and sepsis or sepsis mortality in VLBW Caucasian infants [18] The current study extends those findings by examining a higher risk group (ventilated) of infants with different ethnic backgrounds. In other studies involving primarily Caucasian adults, the CD14 -260 C/T polymorphism does not seem to have a major influence on the risk for or outcome from sepsis [50,51]. Only a single study suggested that the TT genotype (increased CD14) was associated with the development of septic shock and mortality [10]. Increased soluble CD14 concentrations were associated with mortality from Gram-negative septic shock in earlier studies [52].

In Caucasians the CD14 -260 T allele was significantly less frequent in VLBW infants than in term infants, suggesting a potential role for this polymorphism in premature birth. The role of CD14 polymorphisms in prematurity has not been studied but a larger prior study reported by Hartel et al. does not support a role for the CD14 -260CT SNP [53]. The frequency of the CD14 -260 T allele in our control population is similar to that reported in other control Caucasian populations (both adult and term infants), which varied between 0.352 and 0.548 [10,53,54]. The CD14 -260T allele was significantly more frequent in Caucasian control infants than in African-American controls, consistent with other reports [55]. The CD14 -260T allele frequency in African-American infants (both VLBW and term) was similar to that reported in the literature [56,57]. In contrast to that seen in Caucasians, no effect of the CD14 -260CT polymorphism on prematurity was seen in among African-American infants.

An individual's risk for developing sepsis (and its outcome) probably depends on interactions of several genetic factors. Polymorphisms of both pro-inflammatory (IL-6) and anti-inflammatory cytokines (IL-10) may interact either to increase or to decrease risk. In our study, co-carriage of both IL-6 -174 C and IL-10-1082 alleles was associated with a slightly increased risk of late BSI. Multivariate analysis, however, suggests that the IL-10 -1082 A allele is the dominant risk factor.

Other genetic differences may influence risk for and outcome from sepsis in VLBW infants. We recently reported that the TNF-α-308A allele did not affect the risk for sepsis but increased mortality in septic infants [58]. The 3020insC mutation of the NOD2 gene is also associated with increased risk of sepsis in VLBW infants [18]. Polymorphisms in other cytokines, their receptors and other bacterial pattern recognition molecules have been suggested to alter the course of sepsis and are logical candidates for further study in this population [59-62].

The observations of this retrospective case-controlled study are limited by selection bias. Our population of mechanically ventilated infants, most of whom were less than 1000 grams at birth, is at high risk for infection. As a result, the incidence of bacteremia/fungemia in our studied population (47%) is higher than generally reported for a VLBW population and also higher than for the population of VLBW infants in our NICU (approximately 30%) [3]. Mechanical ventilation and lower birth weight are known risk factors for late onset sepsis [63]. Because only infants who were mechanically ventilated were included, the true impact of the polymorphisms studied on the incidence of infectious complications of prematurity may be underestimated.

## Abbreviations

IL-6 Interleukin-6

IL-10 Interleukin-10

VLBW Very Low Birth Weight

CONS Coagulase Negative Staphylococci

BSI Blood Stream Infection

## Competing interests

The author(s) declare that they have no competing interests.

## Authors' contributions

RJB conceived and organized the study, prepared the manuscript and performed the statistical analyses. KY performed the genotyping, and assisted with manuscript preparation. JL oversaw collection tracheal aspirates, assisted with recruitment of subjects into the original studies, and assisted with editing of the manuscript. All authors read and approved the final manuscript.

## Pre-publication history

The pre-publication history for this paper can be accessed here:



## Supplementary Material

Additional File 1 Genotype and allele frequencies of Caucasian and African-American term and preterm infants. Click here for file

Additional File 2 Effects of the IL-10 -1082 GA polymorphisms and infectious complication in Caucasian and African-American infants. Click here for file

Additional File 3 Effects of the IL-10 -1082 GA and IL-6 -174C allele carriage on nosocomial blood stream infections in Caucasian infants. Click here for file

Additional File 4 Effects of the IL-10 -1082 GA and IL-6 -174C allele carriage on nosocomial blood stream infections in African-American infants. Click here for file

Additional File 5 Effects of the IL-10 -1082 GA/IL-6 -174C haplotypes on nosocomial blood stream infections. Click here for file

## Figures and Tables

**Table 1:** Organisms causing Nosocomial Blood Stream Infection, Pneumonia and Endotracheal tube colonization in ventilated VLBW infants

	**CONS**	**Fungi**	**Gram negative bacilli**	***Enterococci *sp.**	***Staphylococcus aureus***	**Other**
Episodes of Nosocomial BSI	113 (56)^a^	33 (16)	26 (13)	9 (4)	6 (3)	14 (7)
ETT Colonization (patients)	136 (46)	27 (9)	39 (13)	27 (9)	14 (5)	30 (10)
Episodes of Nosocomial Pneumonia	1 (5)	7 (32)	10 (45)	0	3 (14)	1 (5)
Sepsis Related Deaths	0	6	5	2	0	2

^a ^Numbers represent the number of episodes of infections (blood stream and pneumonia) or the number of patients with ETT colonization by a particular organism. Numbers in parentheses represent the percentages of total infections (bacteremia and pneumonia) or percentage of all patients (ETT colonization rates). Infants may have been simultaneously or serially colonized with more than one organism, thus the sum of the organism-specific rates is greater than the overall rate of ETT colonization (49%).

CONS coagulase negative staphylococci

ETT endotracheal tube

BSI blood stream infections

**Table 2:** Effect of the IL-6 -174 GC Polymorphism on Nosocomial Blood Stream Infections in African- American and Caucasian Infants

	African American				Caucasian			
Organism	GG (n = 204)	GC (n = 27)	CC (n = 2)	P value	GG (n = 19)	GC (n = 32)	CC (n = 6)	P value

**CONS**	72 (35)	12 (44)	2 (100)	0.116	11 (58)	11 (34)	3 (50)	0.249
**Non CONS**	48 (24)	12 (44)	0	0.185	6 (21)	9 (28)	2 (33)	0.827
Fungal	22 (11)	7 (26)	0	0.071	1 (5)	1 (3)	2 (33)	0.027
Gram negative bacilli	16 (8)	1 (4)	0	0.683	3 (16)	5 (16)	0	0.578
*Enterococcus *sp.	5 (2)	2 (1)	0	0.354	1 (5)	1 (3)	0	0.817
Other	15 (7)	2 (1)	0	0.924	1 (5)	2 (6)	0	0.820

Numbers in parentheses represent percentages of patients with nosocomial blood stream infections

**Table 3:** Effect of the IL-10 -1082 GA Polymorphism on Nosocomial Blood Stream Infections in African- American and Caucasian Infants

	African American				Caucasian			
Organism	GG (n = 27)	GA (n = 118)	AA (n = 88)	P value	GG (n = 13)	GA (n = 25)	AA (n = 19)	P value

**CONS**	8 (30)	45 (38)	33 (38)	0.703	4 (31)	10 (40)	11 (58)	0.276
**Non CONS**	3 (11)	26 (22)	27 (31)	0.088	2 (15)	10 (40)	5 (26)	0.676
Fungal	2 (7)	13 (11)	14 (16)	0.403	0	2 (8)	2 (11)	0.502
Gram negative bacilli	1 (4)	10 (9)	6 (7)	0.675	1 (8)	4 (16)	3 (16)	0.755
*Enterococcus *sp.	0	3 (3)	4 (5)	0.440	0	2 (8)	0	0.265
Other	1 (4)	7 (6)	9 (10)	0.376	1 (8)	2 (8)	0	0.242

Numbers in parentheses represent percentages of patients with nosocomial blood stream infections

**Table 4:** Effect of the CD14 -260 CT Polymorphism on Nosocomial Blood Stream Infections in African- American and Caucasian Infants

	African American				Caucasian			
Organism	CC (n = 109)	CT (n = 101)	TT (n = 23)	P value	CC (n = 23)	CT (n = 24)	TT (n = 10)	P value

**CONS**	34 (31)	39 (39)	13 (57)	0.065	12 (52)	24 (46)	2 (20)	0.224
**Non CONS**	26 (24)	21 (21)	9 (39)	0.178	6 (26)	4 (17)	4 (40)	0.346
Fungal	14 (13)	11 (11)	4 (17)	0.685	2 (9)	1 (4)	1 (10)	0.765
Gram negative bacilli	13 (12)	2 (2)	2 (9)	0.021	3 (13)	2 (8)	3 (30)	0.250
*Enterococcus *sp.	3 (3)	2 (2)	2 (9)	0.229	0	2 (8)	0	0.241
Other	5 (5)	8 (8)	4 (17)	0.095	1 (4)	2 (8)	0	0.592

Numbers in parentheses represent percentages of patients with nosocomial blood stream infections

**Table 5:** IL-10 -1082 A and IL-6 174C Allele Carriage on Nosocomial Blood Stream Infections

	Carriage of IL-10 -1082 A and IL-6 174C Alleles				
	**IL-10 A allele – IL-6 C allele – (n = 31)**	**IL-10 A allele – IL-6 C allele + (n = 9)**	**IL-10 A allele + IL-6 C allele – (n = 195)**	**IL-10 A allele + IL-6 C allele + (n = 58)**	**P value**
**Late BSI (all organisms)**	11 (36)	3 (33)	98 (50)	36 (62)	0.073
**CONS**	10 (32)	2 (22)	41 (36)	12 (44)	0.476
**Non Cons**	3 (10)	2 (22)	23 (20)	10 (37)	0.166
**Multiple BSI**	3 (10)	1 (11)	17 (15)	6 (22)	0.760
**Mortality from sepsis**	1 (3)	0	6 (5)	2 (7)	Na

Numbers in parentheses represent percentages na Not able to calculate
