# Supplementary material for: IL-10, IL-6 and CD14 polymorphisms and sepsis outcome in ventilated very low birth weight infants
Source: BMC Med. 2006 Apr 12;4:10. doi: 10.1186/1741-7015-4-10 (PMC1513390; doi:10.1186/1741-7015-4-10)
Supplement: Additional File 1 — Genotype and allele frequencies of Caucasian and African-American term and preterm infants. [file 1741-7015-4-10-S1.doc]

Supplemental Table 1

Genotype and Allele Frequencies Term vs VLBW infants

|  | African-American | | | Caucasian | | |  |  |
| --- | --- | --- | --- | --- | --- | --- | --- | --- |
|  | VLBW | Term | p value^1^ | VLBW | Term | p value^2^ | p value^3^ | p value^4^ |
| **IL-10 -1082** |  |  |  |  |  |  |  |  |
| GG | 0.116 | 0.149 |  | 0.228 | 0.221 |  |  |  |
| GA | 0.511 | 0.458 |  | 0.439 | 0.516 |  |  |  |
| AA | 0.373 | 0.393 |  | 0.333 | 0.263 |  |  |  |
| A allele | 0.629 | 0.622 | 0.846 | 0.588 | 0.521 | 0.259 | 0.024 | 0.191 |
| **IL-6 -174** |  |  |  |  |  |  |  |  |
| GG | 0.876 | 0.802 |  | 0.333 | 0.448 |  |  |  |
| GC | 0.116 | 0.188 |  | 0.561 | 0.427 |  |  |  |
| CC | 0.080 | 0.01 |  | 0.105 | 0.125 |  |  |  |
| C allele | 0.067 | 0.104 | 0.097 | 0.385 | 0.339 | 0.402 | <0.001 | <0.001 |
| **CD14 -260** |  |  |  |  |  |  |  |  |
| CC | 0.468 | 0.494 |  | 0.404 | 0.183 |  |  |  |
| CT | 0.433 | 0.425 |  | 0.42.1 | 0.524 |  |  |  |
| TT | 0.099 | 0.081 |  | 0.175 | 0.293 |  |  |  |
| T allele | 0.322 | 0.294 | 0.402 | 0.386 | 0.555 | 0.006 | <0.001 | 0.151 |

^1,2^Term vs. VLBW infants

^3^Caucasian vs. African-American infants (Term)

^4^Caucasian vs. African-American infants (VLBW)
