# Supplementary material for: IL-10, IL-6 and CD14 polymorphisms and sepsis outcome in ventilated very low birth weight infants
Source: BMC Med. 2006 Apr 12;4:10. doi: 10.1186/1741-7015-4-10 (PMC1513390; doi:10.1186/1741-7015-4-10)
Supplement: Additional File 2 — Effects of the IL-10 -1082 GA polymorphisms and infectious complication in Caucasian and African-American infants. [file 1741-7015-4-10-S2.doc]

# Supplemental Table 2

Effect of the IL-10 -1082 GA Polymorphism on Infectious Complications in African- American and Caucasian Infants

|  | African American | | | | Caucasian | | | |
| --- | --- | --- | --- | --- | --- | --- | --- | --- |
| Organism | GG  (n=27) | GA  (n=118) | AA  (n=88) | P value | GG  (n=13) | GA  (n=25) | AA  (n=19) | P value |
| Late BSI | 9 (33) | 57 (48) | 48 (55) | 0.153 | 5 (39) | 13 (52) | 13 (68) | 0.235 |
| Multiple BSI | 3 (11) | 18 (15) | 15 (17) | 0.754 | 1 (8) | 5 (20) | 4 (21) | 0.566 |
| Sepsis mortality | 1 (4) | 6 (5) | 4 (5) | 0.950 | 0 | 2 (8) | 2 (11) | 0.502 |
