# Supplementary material for: IL-10, IL-6 and CD14 polymorphisms and sepsis outcome in ventilated very low birth weight infants
Source: BMC Med. 2006 Apr 12;4:10. doi: 10.1186/1741-7015-4-10 (PMC1513390; doi:10.1186/1741-7015-4-10)
Supplement: Additional File 3 — Effects of the IL-10 -1082 GA and IL-6 -174C allele carriage on nosocomial blood stream infections in Caucasian infants. [file 1741-7015-4-10-S3.doc]

Supplemental Table 3

Effect of IL-10 -1082 A and IL-6 -174C Allele Carriage on

Nosocomial Blood Stream Infections (Caucasians)

|  | Carriage of IL-10 -1082 A and IL-6 174C Alleles | | | |  |
| --- | --- | --- | --- | --- | --- |
|  | **IL-10 A allele -**  **IL-6 C allele -**  **(n=5)** | **IL-10 A allele -**  **IL-6 C allele +**  **(n=8)** | **IL-10 A allele +**  **IL-6 C allele -**  **(n=14)** | **IL-10 A allele +**  **IL-6 C allele +**  **(n=30)** | **P value** |
| **Late BSI (all organisms)** | 2 (40) | 3 (38) | 10 (71) | 16 (53) | 0.393 |
| **CONS** | 2 (40) | 2 (25) | 9 (64) | 12 (40) | 0.291 |
| **Non Cons** | 0 | 2 (25) | 4 (29) | 8 (27) | 0.610 |
| **Multiple BSI** | 0 | 1 (13) | 4 (29) | 5 (17) | 0.494 |
| **Mortality from sepsis** | 0 | 0 | 1(7) | 3 (10) | 0.708 |

Numbers in parentheses represent percentage
