# Supplementary material for: IL-10, IL-6 and CD14 polymorphisms and sepsis outcome in ventilated very low birth weight infants
Source: BMC Med. 2006 Apr 12;4:10. doi: 10.1186/1741-7015-4-10 (PMC1513390; doi:10.1186/1741-7015-4-10)
Supplement: Additional File 4 — Effects of the IL-10 -1082 GA and IL-6 -174C allele carriage on nosocomial blood stream infections in African-American infants. [file 1741-7015-4-10-S4.doc]

# Supplemental Table 4

Effect of IL-10 -1082 A and IL-6 174C Allele Carriage on

Nosocomial Blood Stream Infections (African-American)

|  | Carriage of IL-10 -1082 A and IL-6 -174C Alleles | | | |  |
| --- | --- | --- | --- | --- | --- |
|  | **IL-10 A allele -**  **IL-6 C allele -**  **(n=26)** | **IL-10 A allele -**  **IL-6 C allele +**  **(n=1)** | **IL-10 A allele +**  **IL-6 C allele -**  **(n=178)** | **IL-10 A allele +**  **IL-6 C allele +**  **(n=28)** | **P value** |
| **Late BSI (all organisms)** | 9 (35) | 0 | 85 (48) | 20 (71) | 0.031 |
| **CONS** | 8 (31) | 0 | 64 (36) | 14 (50) | 0.371 |
| **Non Cons** | 3 (12) | 0 | 43 (24) | 10 (36) | 0.201 |
| **Multiple BSI** | 3 (12) | 0 | 28 (16) | 5 (18) | 0.789 |
| **Mortality from sepsis** | 1 (4) | 0 | 9 (5) | 1 (4) | 0.974 |

Numbers in parentheses represent percentage
