# Supplementary material for: IL-10, IL-6 and CD14 polymorphisms and sepsis outcome in ventilated very low birth weight infants
Source: BMC Med. 2006 Apr 12;4:10. doi: 10.1186/1741-7015-4-10 (PMC1513390; doi:10.1186/1741-7015-4-10)
Supplement: Additional File 5 — Effects of the IL-10 -1082 GA/IL-6 -174C haplotypes on nosocomial blood stream infections. [file 1741-7015-4-10-S5.doc]

Supplemental Table 5

Effect of IL-10 -1082 GA/ IL-6 -174GC Haplotype on

Nosocomial Blood Stream Infections

|  | **IL-10 -1082GA:IL-6 -174GC Haplotype** | | | | | | | | |
| --- | --- | --- | --- | --- | --- | --- | --- | --- | --- |
|  | **GG/GG**  **(n=31)** | **GG/GC**  **(n=9)** | **GA/GG**  **(n=113)** | **GA/GC**  **(n=27)** | **GA/CC**  **(n=5)** | **AA/GG**  **(n=82)** | **AA/GC**  **(n=23)** | **AA/CC**  **(n=3)** | **P value** |
| **Late BSI (all organisms)** | 11 (36) | 3 (33) | 50 (44) | 18 (67) | 3 (60) | 48 (59) | 13 (57) | 2(68) | 0.122 |
| **CONS** | 10 (32) | 2 (22) | 41 (36) | 12 (44) | 3 (60) | 34 (42) | 9(39) | 2(68) | 0.479 |
| Non Cons | 3 (10) | 2 (22) | 23 (20) | 10 (37) | 0 | 25 (31) | 6 (26) | 2(68) | 0.066 |
| Multiple BSI | 3 (10) | 1 (11) | 17 (15) | 6 (22) | 0 | 15 (18) | 2 (9) | 2(68) | 0.189 |
| Mortality from sepsis | 1 (3) | 0 | 6 (5) | 2 (7) | 0 | 4 (5) | 1 (4) | 1 (33) | 0.512 |

Numbers in parentheses represent percentage
